# An inflammation-related subtype classification for analyzing tumor microenvironment and clinical prognosis in colorectal cancer

**DOI:** 10.3389/fimmu.2024.1369726

**Published:** 2024-04-29

**Authors:** Junpeng Pei, Yuye Gao, Aiwen Wu

**Affiliations:** State Key Laboratory of Holistic Integrative Management of Gastrointestinal Cancers, Beijing Key Laboratory of Carcinogenesis and Translational Research, Unit III, Gastrointestinal Cancer Center, Peking University Cancer Hospital and Institute, Beijing, China

**Keywords:** inflammatory response, colorectal cancer, molecular subtype, prognosis, tumor microenvironment

## Abstract

**Background:**

The inflammatory response plays an essential role in the tumor microenvironment (TME) of colorectal cancer (CRC) by modulating tumor growth, progression, and response to therapy through the recruitment of immune cells, production of cytokines, and activation of signaling pathways. However, the molecular subtypes and risk score prognostic model based on inflammatory response remain to be further explored.

**Methods:**

Inflammation-related genes were collected from the molecular signature database and molecular subtypes were identified using nonnegative matrix factorization based on the TCGA cohort. We compared the clinicopathological features, immune infiltration, somatic mutation profile, survival prognosis, and drug sensitivity between the subtypes. The risk score model was developed using LASSO and multivariate Cox regression in the TCGA cohort. The above results were independently validated in the GEO cohort. Moreover, we explored the biological functions of the hub gene, receptor interacting protein kinase 2 (RIPK2), leveraging proteomics data, *in vivo*, and *in vitro* experiments.

**Results:**

We identified two inflammation-related subtypes (inflammation-low and inflammation-high) and have excellent internal consistency and stability. Inflammation-high subtype showed higher immune cell infiltration and increased sensitivity to common chemotherapeutic drugs, while inflammation-low subtype may be more suitable for immunotherapy. Besides, the two subtypes differ significantly in pathway enrichment and biological functions. In addition, the 11-gene signature prognostic model constructed from inflammation-related genes showed strong prognostic assessment power and could serve as a novel prognostic marker to predict the survival of CRC patients. Finally, RIPK2 plays a crucial role in promoting malignant proliferation of CRC cell validated by experiment.

**Conclusions:**

This study provides new insights into the heterogeneity of CRC and provides novel opportunities for treatment development and clinical decision making.

## Introduction

Colorectal cancer (CRC) is the third most common cancer and the second leading cause of cancer-related death worldwide (1). Treatment of CRC includes radical surgery, supplementary chemotherapy, and/or radiotherapy based on staging and clinico-pathologic disease characteristics. Despite improved screening and treatment over the past few decades, survival of CRC patients remains very poor, with approximately half of patients trapped in recurrence and metastases (2). Patient outcomes and treatment are generally based on clinical stage, although stage-independent factors are also associated with outcomes due to CRC genetic heterogeneity (3). CRC is a heterogeneous disease that evolves through genomic instability due to either microsatellite or chromosomal modifications (4, 5). Inter- or intra-tumor genetic heterogeneity contribute to drug resistance of CRC (6). Thus, the inherent genetic heterogeneity between patients and the urgent need for individualized patient therapy demands intensive exploration of CRC molecular subtypes to ascertain novel biomarkers that permit a more precise prognosis and optimal therapy.

The diverse response to therapy by individual patients may also relate to the tumor microenvironment (TME). Inflammation is a biological process that combines innate and adaptive immune responses with metabolism and various activities that affect cell and organelle integrity and survival (7, 8). It has long been recognized that inflammation has a paradoxical effect on tumors (9–11). Studies have found that TME inflammation occurs either early before tumor detection or remains silent until the late stage of tumor development, supporting the concept that the inflammatory response may function differently during various stages of tumor progression. It is well-accepted that chronic or lingering inflammation is an incubator of carcinoma (12, 13). However, chemokines released during acute inflammation have antitumor effects, with inflammation playing an essential role in the modulation of tumor growth and the adaptive immune response (14). The most representative evidence for a relationship between CRC and inflammation is colitis-associated cancers, specifically ulcerative colitis (UC) and Crohn’s disease (CD), characterized by inflammatory symptoms. However, this relationship accounts for only 1%-2% of all CRCs (15). Colitis-associated colorectal cancer (CAC) is an ‘inflammation-dysplasia-carcinoma’ with DNA mutations induced by an inflammatory response and the release of cytokines by immune cells. While the majority of CRCs are sporadic and formed prior to chronic inflammation. The bioactive molecules produced by cells infiltrating the TME include cytokines, growth factors, and chemokines that contribute to genome instability and immune evasion, promoting tumor progression (16). Thus, understanding inflammation and inflammatory TMEs is essential to the elucidation of the mechanisms of CRC development and improved treatment of CRC. Although many studies have focused on the molecular subtypes of CRC based on gene expression patterns, the study described herein is the first to identify inflammation-related molecular subtypes and to then apply that identification to development of a CRC risk score model.

**Figure 1:**
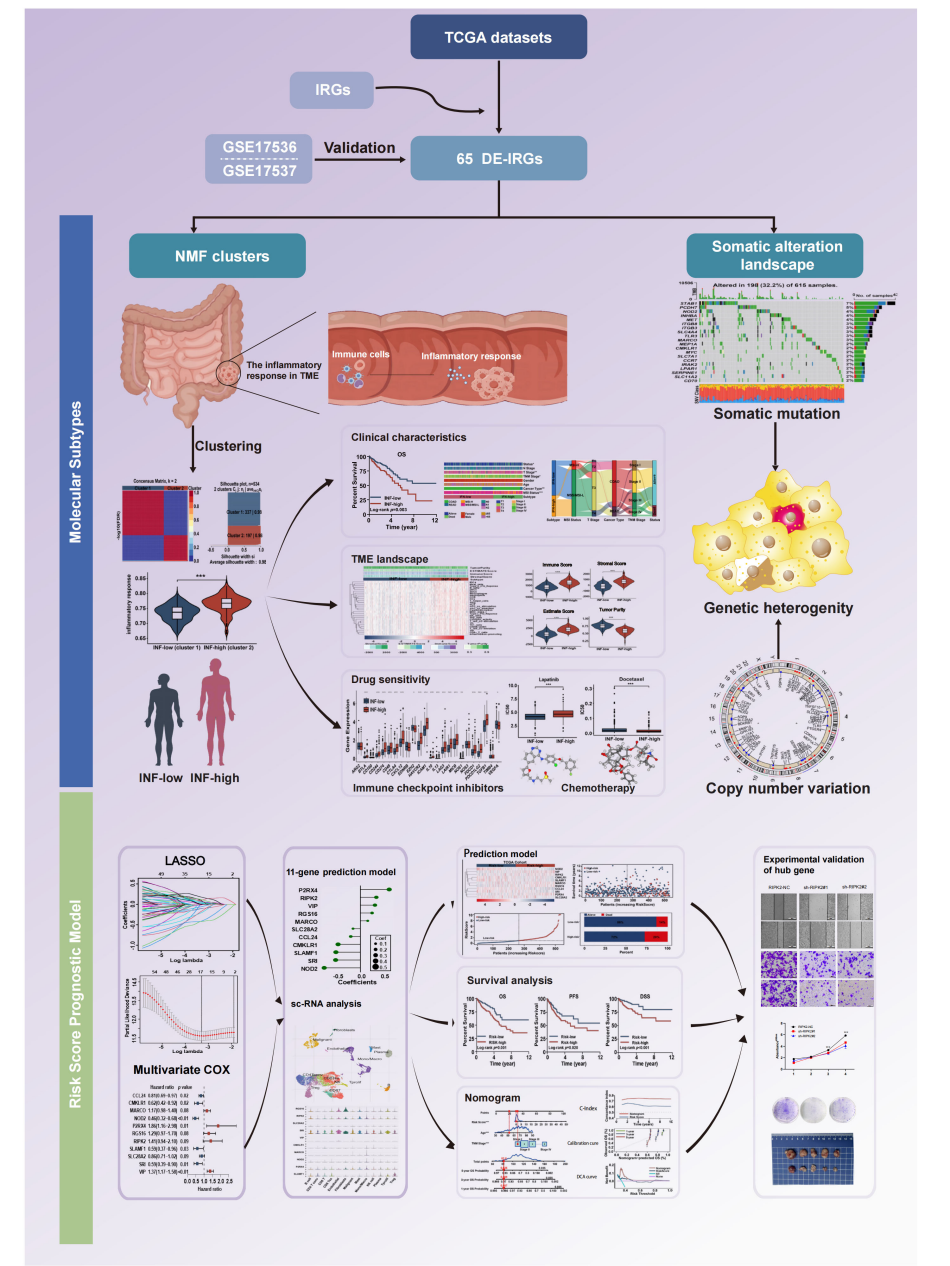
The workflow of designed analysis.

Multiple molecular subtypes of CRC have been identified, the most notable being a consensus molecular subtype (17). The objectives of this study were to identify clinically relevant subtypes of colorectal cancer based on inflammation-related genes. These subtypes were characterized by their clinical prognosis, tumor microenvironment, immune cell infiltration, chemotherapeutic drug sensitivity, and underlying functional mechanisms. Additionally, we aimed to develop a prognostic risk score model and further validate the pivotal signature both *in vivo* and *in vitro*. The workflow of the study is presented in **Figure 1**.

## Materials and methods

### Publicly available cohort data acquisition and preprocessing

Inflammation-related gene sets were collected from the gene-set enrichment analysis (HALLMARK_INFLAMMATORY_RESPONSE). A total of 200 genes served as inflammation-related genes (IRGs) which was shown in **Supplementary Table 1**. Retrieved from The Cancer Genome Atlas (TCGA) database (https://portal.gdc.cancer.gov/), 647 CRC samples and 51 non-cancer samples were evaluated including related TPM RNA-seq data, somatic mutations, and relevant clinical information. Non-cancer refers to the adjacent tissue surrounding colorectal cancer lesions in patients. Microarray data sets of 232 CRC (GSE17538) patients with corresponding clinical information served as an external validation set, acquired from the Gene Expression Omnibus (GEO) database (https://www.ncbi.nlm.nih.gov/geo) (18).

### Identification and mutation landscape of differentially expressed inflammation-related genes

Differentially expressed genes (DEGs) were filtered using the ‘limma’ R package with a |log2fold change (FC)| of > 1 and an adjusted P-value of < 0.05. By intersecting the DEGs in the TCGA dataset, differentially expressed inflammation-related genes (DE-IRGs) were further filtered. The mutation and somatic copy number alterations (SCNA) data of TCGA-COAD and TCGA-READ were downloaded from the Genomic Data Commons using the TCGAbiolinks R package in the MAF format. Using the ‘maftools’ R package, we plotted a waterfall diagram to visualize the mutation landscape of CRC patients. We visualized alteration data with the c-bioportal online tool (https://www.cbioportal.org) (19). Genetic loci was analyzed with the “RCircos”.

### Non-negative matrix factorization, clustering analysis of CRC

Patients with CRC were divided based on the expression of DE-IRGs using the “NMF” R package, considering both survival status and time (20). After selecting the optimum cluster number k, the procedure was iterated 1000 times to allow the construction of a stable and reliable consensus matrix. Silhouette width values varied between −1 and 1. The greater the trend to 1, the greater the degree of separation and cohesion. Principal component analysis (PCA) and t-Distributed Stochastic Neighbor (t-SNE) were performed to show the distribution difference in various inflammation subtypes. The analysis was constructed using the ‘limm’ package and the ‘ggplot2’ package was extensively used to visualize the findings.

### Clinico-pathologic differences of inflammation subtypes

The inflammatory response score of each CRC sample was calculated using single sample Gene Set Enrichment Analysis (ssGSEA) with the aid of the “GSVA” and “GSEABase” packages in R. We subsequently compared the inflammation response scores of Cluster1 and Cluster2 to stratify patient samples into high and low groups. Kaplan–Meier analysis was conducted to compare survival variation between different clusters of different datasets (TCGA and GSE17538) with the aid of the ‘Survminer’ and ‘survival’ packages. For evaluation of the clinical value of the inflammation subtypes, we explored the relationships among inflammation subtypes, prognosis, and other clinico-pathologic features such as age, gender, stage, status, cancer type, and microsatellite instability (MSI) status. The mutual relationships of inflammation subtype and other clinical variables were demonstrated by Sankey diagram using the ‘ggalluvial’ R package. A bar plot was produced to illustrate relationships between clinical traits and biological characteristics of the subtypes. Chi-square tests were performed, and P-values less than 0.05 were considered significant.

### Immune landscape and inflammation subtype mechanisms

The Estimation of Stromal and Immune cells in Malignant Tumor tissues using Expression data (ESTIMATE) method was obtained from the public source website (https://sourceforge.net/projects/estimateproject/) to estimate the stromal score, immune score, tumor purity, and ESTIMATE scores based on specific biomarkers associated with the infiltration of stromal and immune cells in tumor samples (21). ssGSEA was used to measure infiltration levels of immune cell and immune function between different inflammation-related subtypes for each CRC cancer sample.

To assess the biologic basis for immune-related clusters, we conducted Gene Ontology (GO) and Gene Set Enrichment Analysis (GSEA) analyses. GO and GSEA enrichment analyses were implemented using the ‘clusterProfiler’ R package (22). With the use of the reference gene set ‘c2.cp.kegg.v7.5.1.symbols.gmt’ from the MSigDB database (23), we conducted GSEA to identify variations in corresponding pathways among different risk groups (P < 0.05, FDR < 0.25) (24).

### Analysis of drug sensitivity between different inflammation-related groups

We applied a series of predicted indices for response to immune checkpoint inhibitors (ICIs), including immune checkpoints, tumor immune dysfunction and exclusion (TIDE) score, MSI score, tumor mutation burden (TMB), and dysfunction. In this manner relationships among inflammation subtype and the effect of immune therapy were evaluated. RNAss based on mRNA expression and DNAss based on DNA methylation were utilized to measure tumor stemness. The range of scores was from 0 to 1.0, with 1 indicating no differentiation. The ‘pRRophetic’ R package was used to assess semi-inhibitory concentration (IC50) values for chemotherapeutic drugs used for every CRC patient from the TCGA cohort, as a means by which to compare effectiveness for different inflammation subtypes (25).

### Creation and validation of the inflammation-related risk score

The Least absolute shrinkage and selection operator (LASSO) method in the glmnet R package was applied to shrink the scope of gene screening (26). The Cox proportional hazards analysis was used to identify highly inflammation-related genes. Then, the inflammation related risk score (IRRS) was built using the regression coefficients derived from multivariable Cox regression analysis for signatures based on the training set. The risk score formula was calculated as follows:

Risk score = Σ Coefficient of (i)×Expression level of the gene (i). (gene i indicated the identified genes).

The coefficient of gene (i) is the regression coefficient of gene (i), and the expression of gene (i) is the expression value of each candidate IRG (i) for each patient.

The risk score of each patient was determined using the survival R package with the ‘predict’ function. Based on the median value, all patients were divided into high- and low-risk groups. The accuracy of the regression model was commonly tested by Harrell’s Concordance index (C-index). We examined the clinical utility of IRRS through risk score plots and survival curves of the TCGA cohort. We validated the predictive prognosis of IRRS using GEO cohort.

### Establishment of an inflammation-related nomogram

Independent prognostic characteristics, identified by multivariate and univariate Cox analysis of signature, and clinical factors were combined to produce a nomogram using the ‘rms’ R package. Calibration curves of survival probability for different years were plotted using the Hosmer-Lemeshow test. We evaluated the nomogram’s net benefit and clinical utility compared with risk score model based on decision curve analysis (DCA).

### Single-cell RNA sequence analysis

To examine single-cell RNA sequence (scRNA-seq) data collected from GSE146771, tumor immune single-cell hub (TISCH) analysis was employed (27). TISCH is a single-cell RNA-seq data source that puts an emphasis on the TME and provides specific annotation of cell types at the single-cell level, allowing for TME investigation across diverse malignancies (28).

### Validation of hub gene expression patterns

Using TRIzol reagent (#15596018, Invitrogen, Carlsbad, CA, USA) to isolate and extract total RNA, reverse transcription was conducted using a PrimeScript™ RT Reagent Kit (Cat#: E096-01A, Novoprotein, Shanghai, China). Based on the TB Green Premix Ex Taq (Novoprotein, Inc.) protocol, specific primers were used to perform quantitative real-time PCR. GAPDH was used as an internal control, and the 2^-ΔΔCt^ method was used to calculate relative mRNA levels. Technical and biological replicates of each gene were performed at least three times during RT-qPCR analysis. **Supplementary Table 2** contains the RNA molecules evaluated on cell lines and their corresponding primers.

### Cell culture, immunohistochemistry and stable transfection of shRNA

Detailed information on cell culture, IHC and transfection are described in the article/**Supplementary Material**.

### Cell proliferation and plate clone formation assays

A total of 5000 transplanted cells per well were cultured in 96-well plates, with each well incubated in 100 μl of culture media. Six parallel wells were maintained for each experimental group. At specified intervals, 90 μl of culture medium and 10 μl of CCK8 solution (Biosharp, China) were introduced to replace the existing media. Following a 1.5-hour incubation period, absorbance at 450 nm was determined using a microplate reader.

For the colony formation assay, 500 cells per well were inoculated into 6-well plates and cultured for a duration of 10 days. After fixing the colonies with 4% formaldehyde and staining them with a 0.5% crystal violet solution, images were captured.

### Wound healing assay

Various cell groups, including the RIPK2-NC and sh-RIPK2 groups, were collected and seeded in 6-well plates at a density of 1×10^6^ cells/well. The cells were cultured in antibiotic-free medium until they reached 90% confluence. Subsequently, a linear wound was meticulously created in the confluent monolayer using a pipette tip and ruler. After the removal of floating cells with PBS, serum-free medium was introduced. The wounds were promptly photographed at time 0 hours and subsequently at the 48-hour mark. The cells were cultivated in a 37°C, 5% CO2 incubator throughout the experiment. Wound size was assessed at 5 random sites perpendicular to the wound. The wound healing rate (%) was calculated as (0 h scratch area - 48 h scratch area)/0 h scratch area. Each experiment was meticulously replicated a minimum of 3 times.

### Transwell assay

Cell migration and invasion were assessed using a sterile 6.5 mm Transwell equipped with an 8.0 μm pore polycarbonate membrane insert (#3422, Corning, Cambridge, MA, USA). For the migration experiment, 200 µl of cell suspension containing 3×10^4^ cells were introduced into the upper chamber, while 700 µl of medium containing 20% FBS was added to the lower chamber. Each experimental group was cultured in an incubator with 3 wells. Following 36 hours of incubation at 37°C and 5% CO2, cells were carefully extracted from the chamber. The medium in the upper chamber was discarded, and the upper chamber cells were gently wiped off using a cotton swab. Cells that migrated to the lower surface of the filter were fixed with 4% paraformaldehyde for 15 minutes and subsequently stained with a 0.1% crystal violet solution for 10 minutes. After washing the cells with PBS three times, cell counts were conducted in 5 random visual fields per insert under a 20-fold inverted microscope, and photographs were taken. For the invasion experiment, a 50 mg/L Matrigel solution was diluted 1:4 in serum-free medium. The bottom membrane of the Transwell chamber was coated with the Matrigel mixture, air-dried at 4°C, and hydrated. The subsequent steps mirrored those in the migration experiment.

### *In vivo* animal studies

BALB/c nude mice (6-8 weeks of age, male, 24-28 g) were procured from Beijing HFK Bio-technology Co. Ltd. (Beijing, China). The mice were housed in a specific pathogen-free barrier facility and were utilized in strict accordance with protocols approved by the Institutional Animal Care and Use Committee (IACUC) at the Peking University Cancer Hospital. The BALB/c nude mice were randomly allocated into 2 groups (n = 5/group). SW620 cells, transfected with RNA lentivirus (Genomeditech, Shanghai, China), were collected, washed, and resuspended in serum-free medium at a concentration of 5×10^7^ cells/ml. For tumorigenesis studies, each mouse (5 in each group) was injected with 5×10^6^ SW620 cells stably transfected with the indicated expression vectors, suspended in 100μL PBS, in the left axilla. Tumor measurements were conducted every 3 days using the formula: volume =1/2 × width^2^ × length. After a 4-week period, the mice were humanely sacrificed, tumors were harvested, weighed, and sent for subsequent analysis.

### Statistical analysis

Bioinformatics analyses were completed using R software (version 4.2.1). A two-sided P-value was used, with results considered statistically significant when the P-values were less than 0.05. Survival disparities between groups were examined by drawing Kaplan-Meier curves, with differences in survival estimated by the two-sided log-rank test. Divergence between two groups was evaluated by the Wilcox test.

## Results

### Somatic alteration landscape of inflammation-related genes

**Figure 2:**
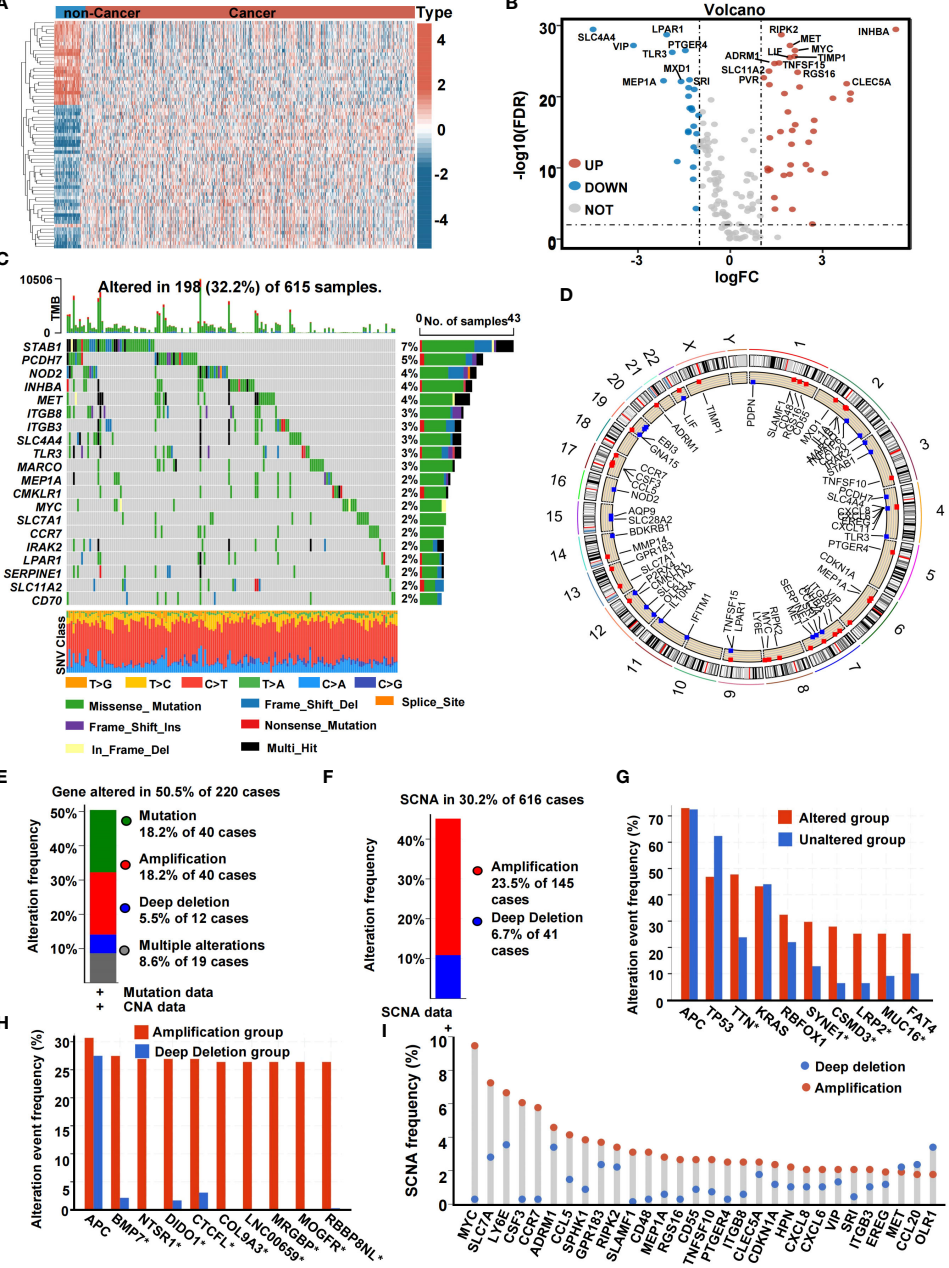
The genetic landscape of IRGs in CRC. **(A)** Heatmap of the expression of IRGs in non-cancer samples and cancer samples. **(B)** Volcano plot of IRGs. **(C)** Landscape of genomic aberrations of the IRGs in CRC. Each row represents a gene, and each column represents a patient. The frequency of alterations in top 20 IRGs is displayed on the right side of the mutation landscape. **(D)** The localization of the SCNA. **(E)** Histogram of the proportion of gene alteration in CRC. **(F)** Histogram of the proportion of SCNA in CRC. **(G)** Gene alteration frequency of CRC patients in TCGA. **(H)** SCNA frequency of CRC patients in TCGA. **(I)** Lollipop chart of the SCNA proportion in IRGs (*,<0.05).

We extracted the SCNA and mutation spectrum of the 647 CRC patients from the TCGA cohort. Inflammation genes of the 647 CRC samples and 51 non-cancer samples were evaluated. Differential analysis of IRGs between CRC and normal non-cancer tissue yielded 65 DE-IRGs. Among these, 25 were found to have poor expression in tumor tissue and 40 were found to have high expression in tumor tissue (**Figures 2A, B**). We analyzed the genetic variation of CRC-related inflammation genes, including the top 20 mutated genes. Of the 615 (32.2%) samples, 198 were mutations, of which missense mutations were the most common. DE-IRGs with the highest mutation frequency were *STAB1* (7%), *PCDH7* (5%), *NOD2* (4%), *INHBA* (4%), and *MET* (4%) (**Figure 2C**). We identified the locus of SCNA hotspots on chromosomes in **Figure 2D**. Based on **Figure 2E**, genes were altered in 50.5% of 220 cases. SCNA accounted for most DNA alterations. As shown in **Figure 2F**, among CRC patients, 30.2% had at least one SCNA of IRGs. **Figure 2G** shows the gene alteration frequency of CRC patients. **Figure 2H** shows the SCNA frequency of CRC patients. Among the inflammation genes, most inflammation genes with high CNA frequency trended to result from co-amplification rather than co-deletion (**Figure 2I**).

### NMF clustering identified two inflammation-based subtypes

**Figure 3:**
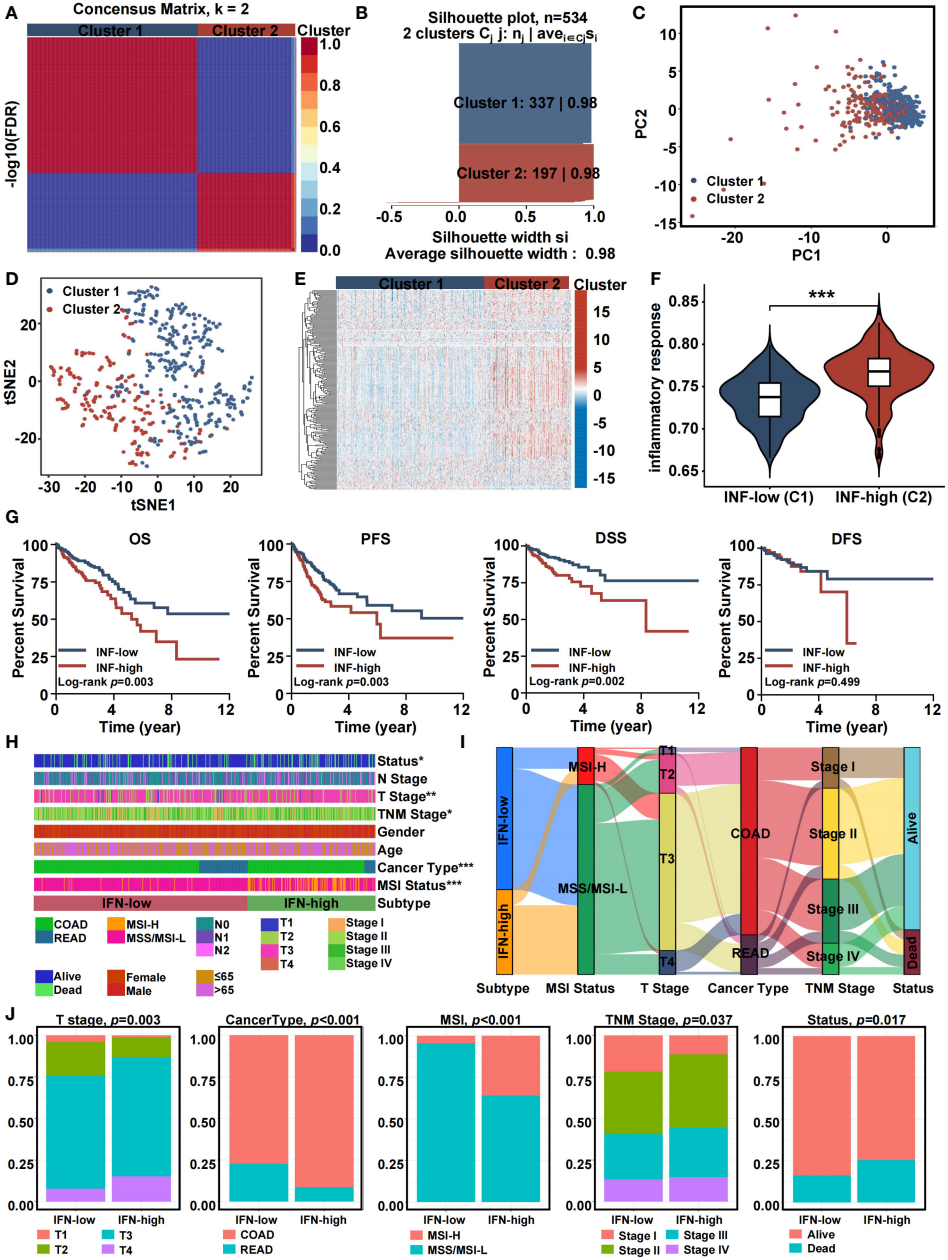
Consensus clustering of inflammation-related genes in training cohort. **(A)** The consensus clustering heat map visualizes the degree of segmentation for 65 genes in 534 samples. **(B)** The average silhouette width represents the coherence of clusters. **(C)** Principal component analysis plots. **(D)** t-SNE plots. **(E)** Heatmap of 65 inflammation related genes expression in different subgroups; red represents high expression, and blue represents low expression. **(F)** Violin plots indicating the differences in these subtypes. **(G)** Kaplan-Meier overall survival, progress-free survival, disease-specific survival, and disease-free survival curves. **(H)** Heatmap presenting the clinicopathologic features of these subtypes. **(I)** Sankey diagram showing the relationship between inflammation subtype, MSI status, T stage, cancer type, TNM stage and status. **(J)** The distribution characteristics of different clinicopathological factors in two subtypes. (*p<0.05, **p<0.01, and ***p<0.001).

Based on the completeness of clinical information, a total of 534 patients were included in the NMF clustering. Based on the expression profiles of DE-IRGs from TCGA, the NMF algorithm was used to cluster patients into two different expression patterns, Cluster 1 (n = 337) and Cluster 2 (n = 197) (**Figure 3A**). As shown in **Figure 3B**, when k = 2, the two subtypes had a clear boundary, suggesting a high degree of explanation and interpretation of the cluster with extremely strong intra-cluster and low inter-cluster correlation. PCA was conducted to compare the transcriptional profiles of both inflammatory subtypes. In general, PCA and t-SNE indicated that patients could be divided into two remarkably different subtypes with distinct transcriptional profiles (**Figures 3C, D**). The expression landscape of DE-IRGs varied among other clusters. Specifically, Cluster 1 had a lower level of inflammation gene expression. In contrast, Cluster 2 was found to have a higher expression level of inflammation genes (**Figure 3E**). The inflammatory response score of each patient was quantified by the ssGSEA method. **Figure 3F** demonstrates that patients stratified into Cluster 2 have a greater inflammatory response compared to patients in Cluster 1. Hence, we designated Cluster 1 as an inflammation-low (INF-low) subtype, and Cluster 2 as an inflammation-high (INF-high) subtype. We validated the inflammation-based classification in an independent sample cohorts (GSE17538) (**Supplementary Figure 1**).

Patients classified into the different inflammation subtypes had different survival outcomes and clinico-pathologic features. In general, the INF-high subtype had a dismal prognosis with a shorter overall survival time (OS), progress-free survival (PFS), and disease-specific survival (DSS) (log-rank test, P ≤ 0.05) (**Figure 3G**). The relationship between clinico-pathologic characteristics of the two TCGA subtypes are shown in **Figures 3H-J**. Patients in the INF-high group had greater mortality, a T3-T4 stage, a stage II-IV, colon adenocarcinoma cancer type, and microsatellite-instability status. The Sankey plot intuitively presented the relationships between the inflammation-related subtypes, MSI status, T stage, cancer type, TNM stage, and the status of CRC patients. Using GSE17538 cohort, we conducted external validation to confirm clustering resilience (**Supplementary Figure 1**).

### Inflammation-based subtypes are associated with distinct tumor microenvironments

**Figure 4:**
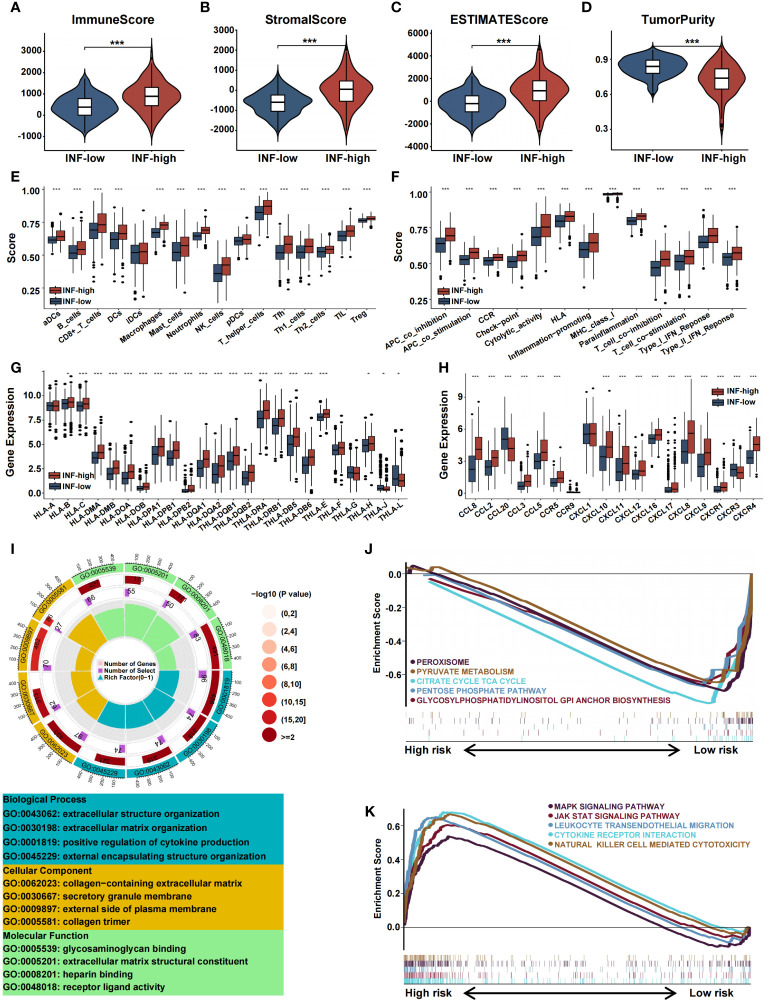
Immune landscape of inflammation subtypes in the training cohort. **(A-D)** The violin plots display the immune score, stromal score, estimate score, and tumor purity score in the training cohort. **(E-H)** Boxplots representing the differential expression of immune cells, immune cell subpopulations related functions, HLA gene sets and chemokines. **(I)** GO analysis of DE-IRGs in terms of biological process, cellular component and molecular function. **(J, K)** GSEA of the DE-IRGs showing the different pathways in the INF-low group and in the INF-high group. (*p<0.05, **p<0.01, and ***p<0.001).

The TME component was explored using the ESTIMATE algorithm, which showed that the INF-high group had higher stromal scores, ESTIMATE scores, immune scores, and lower tumor purity (P < 0.05) (**Figures 4A-D**). The TME component was related to inflammation and played a decisive role in immune cell infiltration. By using ssGSEA, we assessed the immune function of the two TCGA subtypes. Compared with the INF-low subgroup, the INF-high subgroup exhibited a greater immune cell infiltration and more active levels of immune-associated processes and pathways, indicating a microenvironment of excessive immune activation (**Figures 4E, F**). The relationship of the two subtypes to major histocompatibility complexes was assessed. The expression level of the major histocompatibility complexes tended to be higher in the INF-High group (**Figure 4G**). The immunological profile was assessed by analysis of inflammatory markers. The concentration of multiple chemokines was markedly increased in the serum of INF-high patients compared to INF-low patients, which indicates a more severe inflammatory response in the INF-high subgroup (**Figure 4H**). The immune landscape of inflammation subtypes in the validation cohort is presented in **Supplementary Figures 2A-J**. The somatic variations in CRC driver genes between the INF-high and INF-low subgroups is shown in **Supplementary Figures 2K, L**.

We performed functional enrichment analysis of IRGs to gain insight into the potential role of inflammation-related genes and their relationships with the CRC immune microenvironment. GO analysis showed that IRGs are associated with biological processes such as extracellular structure organization, extracellular matrix organization, positive regulation of cytokine production, and external encapsulating structure organization. We performed enrichment analysis of the two inflammation clusters to determine pathways involved the regulation of tumorigenesis. GSEA analysis demonstrated IRGs to be highly expressed in the INF-high group with significant enrichment in MAPK signaling, JAK-STAT signaling, leukocyte trans-endothelial migration, cytokine receptor interaction, and natural killer cell-mediated cytotoxicity. In contrast, the INF-low subtype was substantially enriched in negative modulation of cell metabolism pathways, including peroxisome, pyruvate metabolism, citrate cycle TCA cycle, pentose phosphate pathway, and glycosylphosphatidylinositol anchor biosynthesis (**Figures 4I-K**).

### Prediction of chemotherapy and immunotherapy sensitivity

**Figure 5:**
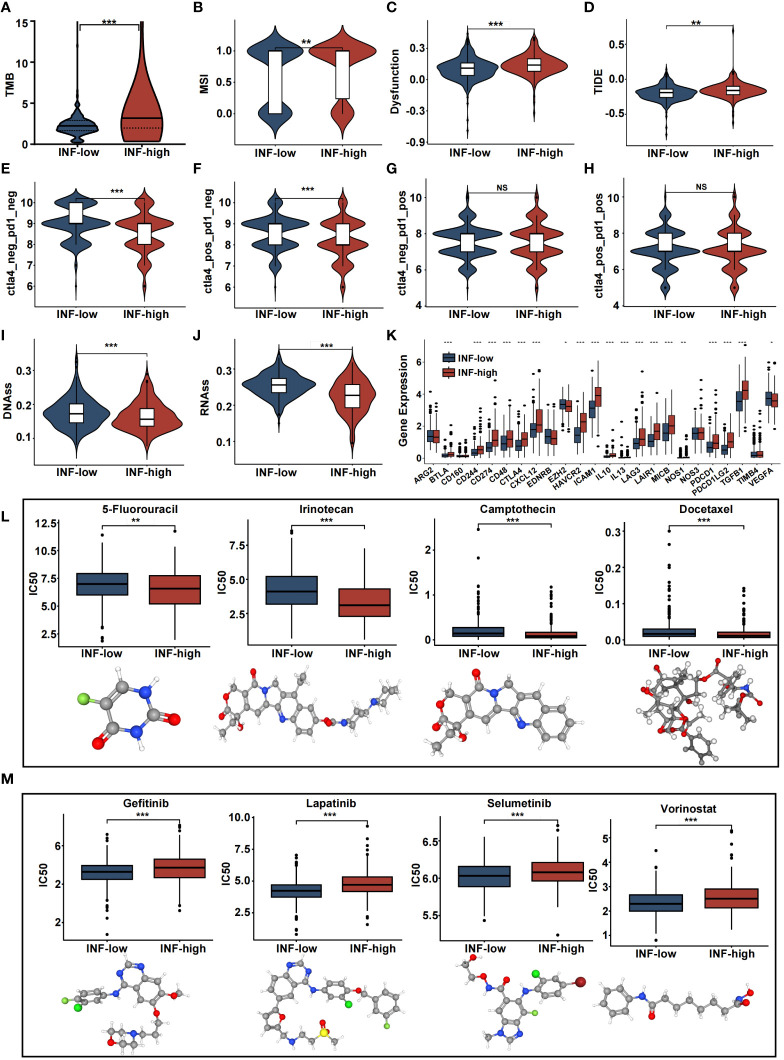
The estimation of two inflammation subtypes in immunotherapy and chemotherapy response. **(A-J)** Violin plots presenting the TMB score, MSI score, dysfunction score, TIDE score, IPS, IPS-CTLA4, IPS-PD1, IPS-PD1-CTLA4 scores, DNAss and RNAss between two IRRS subtypes. **(K)** Boxplot representing the immune checkpoints in the various inflammation subtypes in the various inflammation subtypes. **(L, M)** drug sensitivity analysis and 3D structure tomographs of the eight candidate common drugs for CRC. (NS, no significance; *p<0.05, **p<0.01, and ***p<0.001).

Inflammation gene profiles were assessed with regard to treatment choice. Results showed that the INF-high subgroup patients had higher TMB values, dysfunction scores, and MSI scores (**Figures 5A-C**). The patient’s response to immune checkpoint inhibitors and survival were negatively related to the TIDE score. The TIDE score of INF-high patients was significantly higher than that of INF-low patients (P < 0.05), indicating that INF-high patients had a poor response to immune checkpoint inhibitors, poorer survival, and a tendency to immune escape (**Figure 5D**). We utilized the IPS as a means by which to assess the response to immune checkpoint inhibitors. The scores for IPS and IPS-CTLA4 blockers were lower for the INF-high group, indicating that the INF-high group had a poorer result with immunotherapy (**Figures 5E-H**). These findings indicate that the low inflammation subtype was significantly related to a better immunotherapy effect and that INF-high patients exhibit an immune-suppressive microenvironment. Tumor stemness was assessed among the different CRC subtypes by RNAss and DNAss and results showed a negative effect of the INF-high subtype on cancer cell characterization (**Figures 5I, J**). Further, the expression levels of immune checkpoint molecules including BTLA, CTLA-4, and LAG-3 were significantly upregulated in the high inflammatory response group compared with the low inflammatory response group, indicating a higher tendency to escape from host immunity of INF-high subgroup (P < 0.05) (**Figure 5K**).

Chemotherapeutic efficacy and clinical utility, of the inflammation-related classification, were assessed with regard to precise CRC treatment. The results showed that many common CRC chemotherapy drugs had significantly different effects on the INF-high groups of patients (P < 0.05). 5-Fluorouracil (5-FU) is the most common chemotherapy drug for CRC and had a significantly lower IC50 value for the high-INF group than for the low-INF group (P < 0.05). Of note, INF-high patients were more sensitive to 5-FU, Camptothecin, Irinotecan, and Docetaxel. Further, INF-low individuals responded more to non-traditional drugs such as Lapatinib, Selumetinib, Vorinostat, and Gefitinib (P < 0.05). These results suggest that classification based on inflammatory response may be an effective means to predict drug response and potential therapeutic targets (**Figures 5L, M**). 3D structures were mapped for chemotherapeutic agents with differences between the two groups, using the PubChem database.

### Development and validation of the CRC predictive model

**Figure 6:**
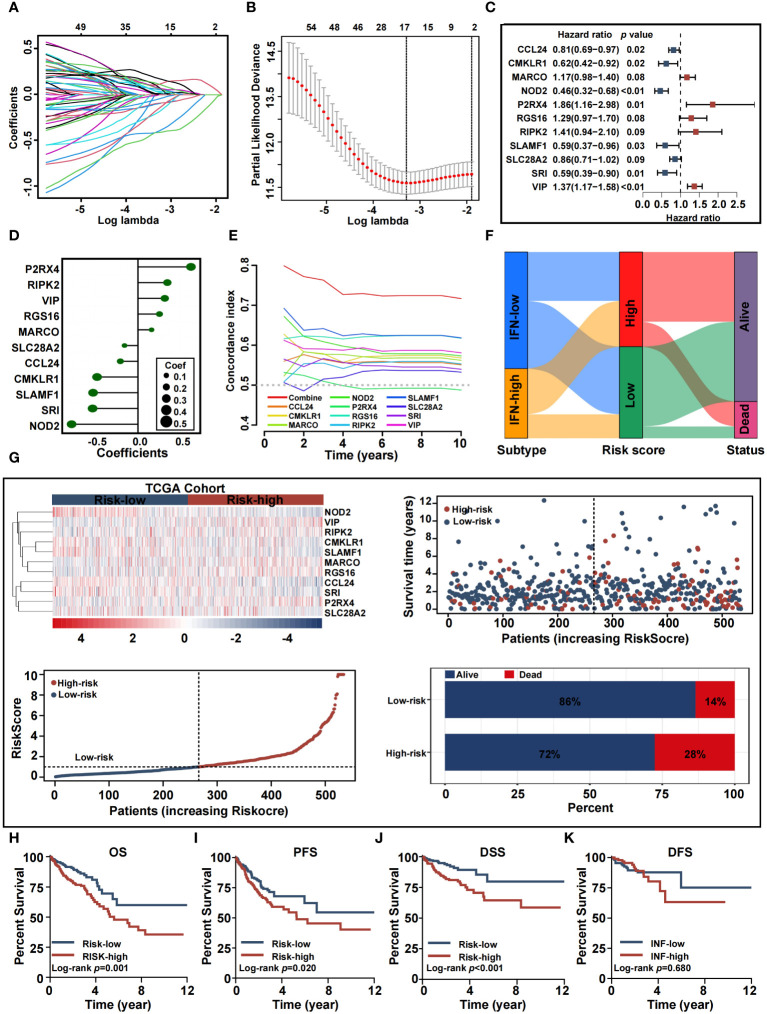
Construction and validation of the inflammation-related prognostic signature in training cohort. **(A, B)** Lasso analysis of 65 inflammation genes associated with overall survival. **(C)** Multivariable Cox analysis uncovered 11 inflammation genes associated most with overall survival. **(D)** The coefficient of the 11 genes identified by Multivariable Cox analysis. **(E)** Time-dependent C-index plot for the risk score and individual genes. **(F)** Sankey plot summarized the relationships among the clusters, IRRS and survival status. **(G)** Risk scores distribution, survival status of each patient, and heatmaps of prognostic eleven-gene risk signature. **(H-K)** Kaplan−Meier analysis of overall survival, progress-free survival, disease-specific survival, and disease-free survival curves in the two risk groups.

We constructed a predictive model based on the 647 CRC patients, with OS information, derived from the TCGA cohort. Through LASSO COX regression analysis, we chose and evaluated DE-IRGs significantly associated with survival. Seventeen genes were selected to build a predictive model using the optimal adjustment parameter (λ) via the LASSO method (**Figures 6A, B**). After univariate COX proportional hazards analysis, 11 genes (*P2RX4, RIPK2, VIP, RGS16, MARCO, SLC28A2, CCL24, CMKLR1, SLAMF1, SRI*, and *NOD2*) met the proportional hazard hypothesis and were used to establish the risk score model. We drew a forest plot to demonstrate the associations between the expression levels of the 11 inflammation-related signatures and OS (**Figure 6C**). The expression levels of *P2RX4, RIPK2, VIP, MARCO*, and *RGS16* had significant positive contribution to a better prognosis, while the expression levels of *SLC28A2, CCL24, CMKLR1, SLAMF1, SRI*, and *NOD2* had an opposite effect. Calculation of the risk-score model was achieved by the following equation: risk score = (0.6184) × *P2RX4* + (0.3427) × *RIPK2* + (0.3129) × *VIP* + (0.2508) × *RGS16* + (0.1581) × *MARCO* + (−0.1568) × *SLC28A2* + (0.2054) × *CCL24* + (−0.4771) × *CMKLR1*+ (−0.5236) × *SLAMF1*+ (−0.5293) × *SRI* + (−0.7741) × *NOD2* (**Figure 6D**). We conducted time-dependent C-index curves of different factors, with the combined model having the highest C-index compared to single variables (**Figure 6E**). Patients were assigned to high-risk and low-risk cohorts by median-risk score. In **Figure 6F**, the Sankey plot shows the relationships between inflammation-related subtypes, IRRS, and survival status. The high IRRS group had a more significant proportion of patients with a deadly outcome, while the INF-low group had a higher proportion of patients with low IRRS. Relationships between risk score distribution and survival status were examined. **Figure 6G** presents survival outcomes, IRRS, and model gene expression profiles between the two risk groups. Kaplan–Meier survival analysis verified that the high-risk TCGA cohort was associated with poorer OS, PFS, and DSS, while the low-risk TCGA cohort was not (**Figures 6H-K**). These results were validated with the GEO cohort (**Supplementary Figure 3**).

### Establishment of an inflammation-based nomogram

**Figure 7:**
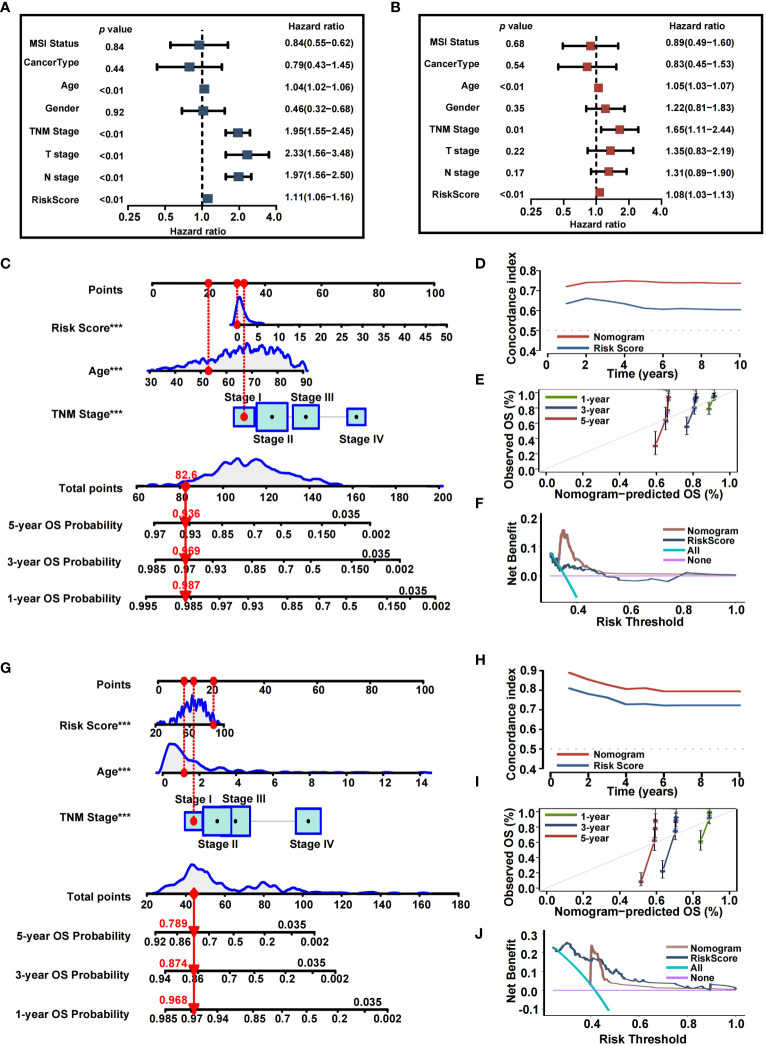
Nomogram developed for predicting the clinical outcome. **(A, B)** Univariate analysis and multivariate analysis containing IRRS and clinical factors. **(C)** The comprehensive nomogram for predicting probabilities of CRC patients with 1-, 3- and 5-year OS in TCGA dataset. **(D)** Time-dependant c-index plot for the nomogram and other clinical factors in the TCGA cohort. **(E)** The calibration plots for predicting CRC patients with 1-, 3- and 5-year OS in the TCGA cohort. **(F)** Decision curve analysis of the nomogram and other factors in the TCGA cohort. **(G)** The comprehensive nomogram for predicting probabilities of CRC patients with 1-, 3- and 5-year OS in GEO dataset. **(H)** Time-dependant C-index plot for the nomogram and other clinical factors in the GEO cohort. **(I)** The calibration plots for predicting CRC patients with 1-, 3- and 5-year OS in the GEO cohort. **(J)** Decision curve analysis of the nomogram and other factors in the GEO cohort.

Since high IRRS was significantly associated with higher malignancy and advanced CRC tumor, we sought to determine whether IRRS was a clinically independent prognostic factor for CRC patients. IRRS and significant clinico-pathological indicators were subjected to univariate and multivariate Cox analysis (**Figures 7A, B**). After adjustment for potential bias, multivariate regression analysis demonstrated age (1.05, 1.03−1.07, P < 0.01), TNM stage (1.65, 1.11−2.44, P < 0.01), and IRRS (1.08, 1.03−1.13, P < 0.01) to be independent factors that predicted prognosis for CRC patients. Based on the above results, we established a comprehensive nomogram that acts as a clinically relevant quantitative tool by which clinicians can predict the 3-, 5-, and 10-year OS probabilities for CRC patients (**Figure 7C**). Every patient was assigned a total point value by addition of the values for each prognostic parameter. Higher total points corresponds to worse patient outcomes. Time-dependent C-index curves of the nomogram and risk score, based on TCGA, demonstrated the nomogram to have better performance and more accurate survival prediction that the risk score model (**Figure 7D**). Furthermore, calibration plots of the TCGA cohort indicated that the nomogram had high consistency between predicted and actual OS (**Figure 7E**). To explore the clinical applicability of the nomogram, we generated a DCA curve that showed the comprehensive nomogram to have more net benefit than a model with risk score (**Figure 7F**). The predictive value of the nomogram was verified with the GEO cohort (**Figures 7G-J**).

### Validation of inflammation gene expression patterns by scRNA-seq analysis

**Figure 8:**
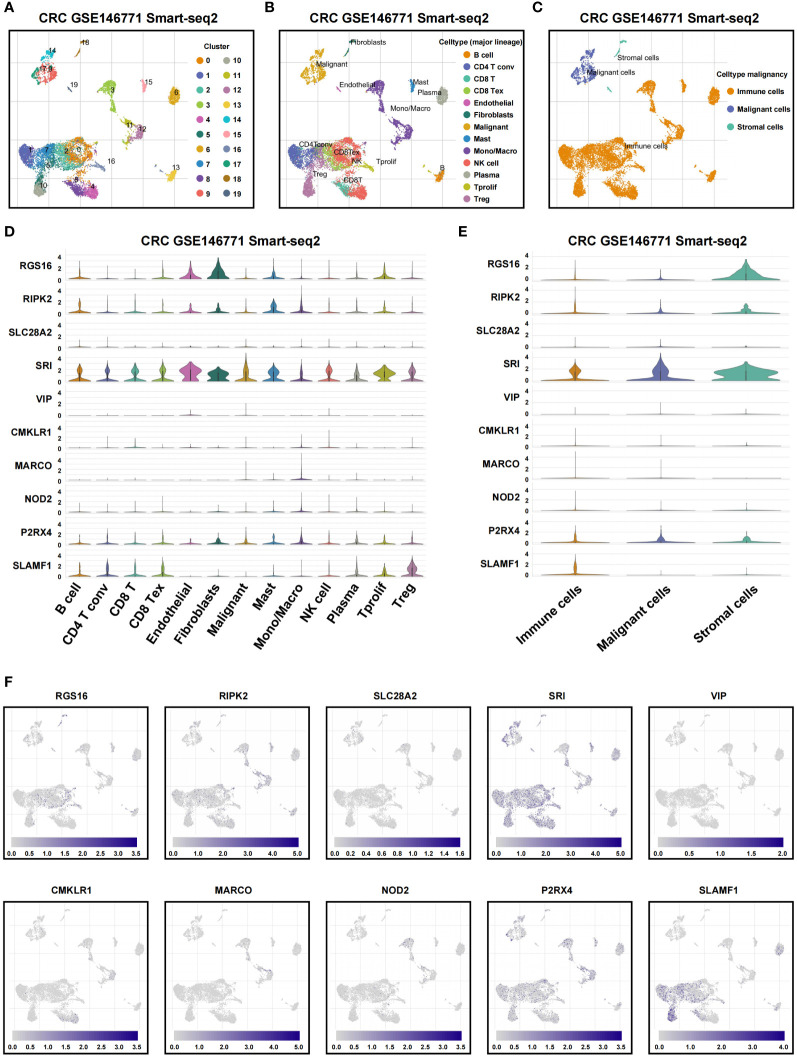
Single-cell profiles reveal inflammation genes expression patterns. **(A)** Cells were clustered into 20 types by the UMAP dimensionality reduction algorithm, with each color representing an annotated phenotype. **(B)** UMAP plot of 13 predominant cell types from colon cancer scRNA-seq data. **(C)** UMAP plot of immune, malignant and stromal cells from colon cancer scRNA-seq data. **(D, E)** Violin plot for displaying the expression levels of inflammation genes in all cell types. **(F)** UMAP plots for visualizing the abundance distribution of inflammation genes.

To assess associations among TME cell types and the expression of signatures in IRRS, we analyzed GSE146771 CRC scRNA-seq data. 20 cell clusters were delineated using uniform manifold approximation and projection (UMAP)-based cell clustering after dimensionality reduction (**Figure 8A**). B cell, conventional CD4+ T cells (CD4Tconv), CD8T, exhausted CD8+ T cells (CD8Tex), endothelial cells, fibroblasts, malignant cells, mast cells, NK cells, monocytes or macrophages, plasma, Tprolif cells, and Treg were labeled based on lineage markers (**Figure 8B**). The 20 clusters were merged into three cell types that included immune cells, malignant cells, and stromal cells (**Figure 8C**). Results showed that *P2RX4, RIPK2* and *SRI* were predominantly expressed in most cell types; *SLAMF1* was predominantly expressed in immune cells; *RGS16* was mainly expressed in stromal cells. *SLC28A2, VIP, CMKLR1, MARCO*, and *NOD2* were detected at low levels in non-tumor and tumor cells (**Figures 8D-F**). *CCL24* was not detected at the single cell level.

### Inflammation pathway hub gene identification

**Figure 9:**
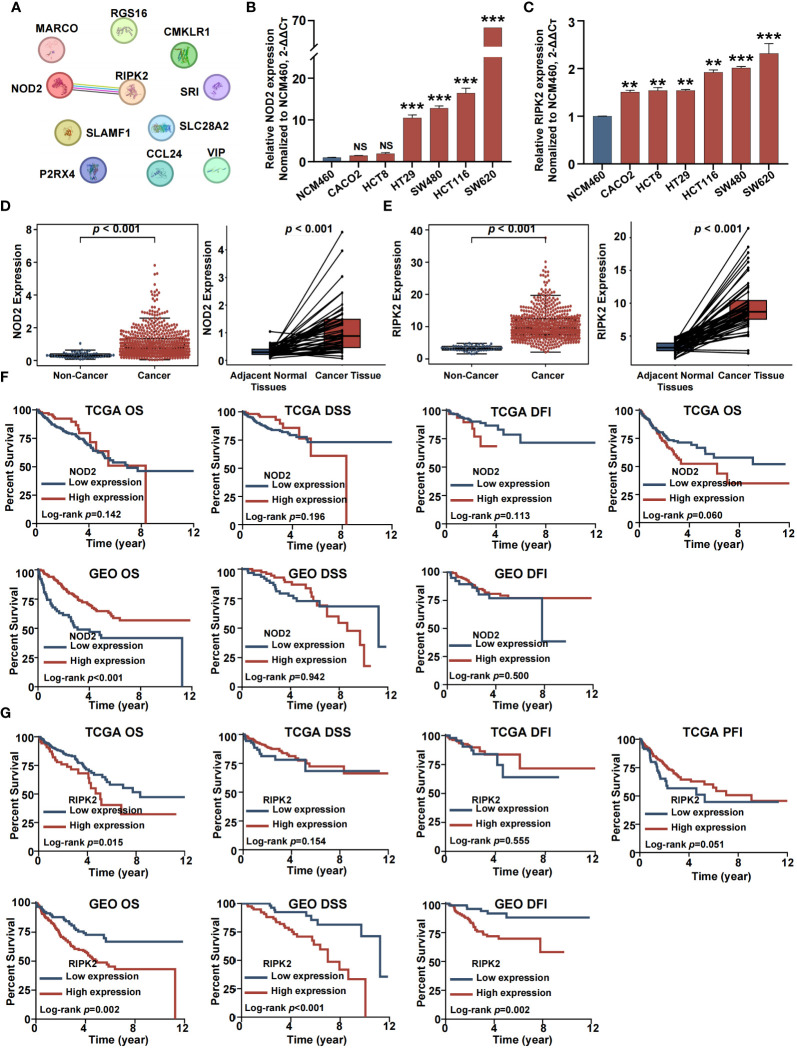
Identification of two hub inflammation genes in CRCs. **(A)** Protein–protein interactions among 11 inflammation related genes. **(B)** RT-QPCR analysis of NOD2. **(C)** RT-QPCR analysis of RIPK2. **(D)** Differential analysis for NOD2. **(E)** Differential analysis for RIPK2. **(F)** Survival analysis for NOD2. **(G)** Survival analysis for RIPK2. (NS, no significance; *p<0.05, **p<0.01, and ***p<0.001).

For experimental verification, the number of inflammation-related genes was narrowed by construction of a protein-protein interaction (PPI) network. This was accomplished by exploration of the STRING database for hub genes within the 11 genes that comprised the inflammation-related risk score system. As shown in **Figure 9A**, there was a complex interaction between *RIPK2* and *NOD2*, suggesting that these were central nodes of the PPI network. Thus, we chose both genes for further study. *NOD2* and *RIPK2* were expressed differentially by tumor and normal or adjacent normal tissue. RT-qPCR demonstrated that CRC cells showed higher mRNA expression of *NOD2* and *RIPK2* compared with control cells (**Figures 9B, C**). Moreover, both genes were upregulated in cancer tissue and significantly associated with CRC clinical outcome based on GEO and TCGA cohorts (**Figures 9D-G**).

### RIPK2 promotes malignant proliferation of CRC cells

**Figure 10:**
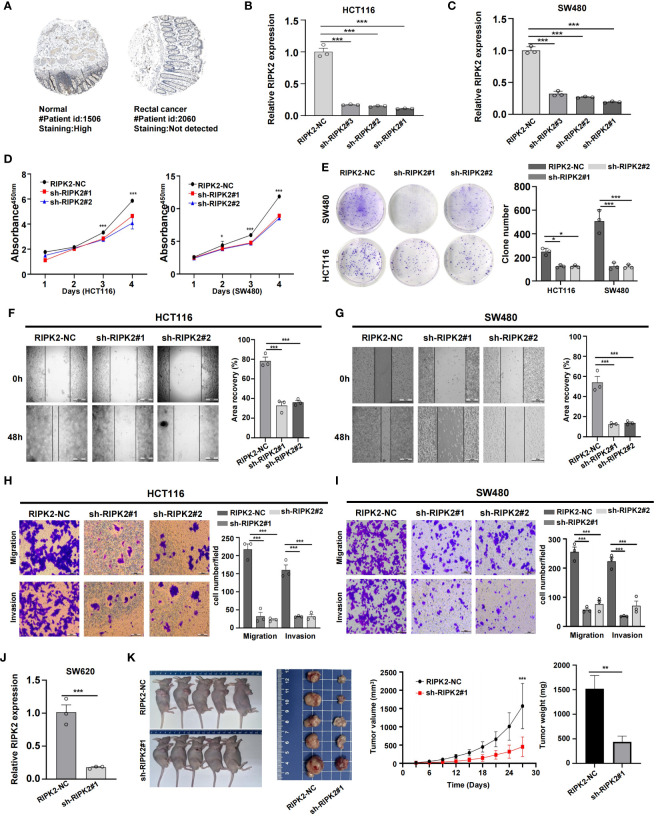
RIPK2 promotes malignant proliferation of CRC cells. **(A)** Distribution of protein expression in rectum and rectal cancer tissues obtained from the HPA database. **(B)** Differential expression of RIPK2 proteins. **(C)** Efficiency of RIPK2 knockdown in HCT116 and SW480 cells. **(D)** CCK-8 assay measuring cell viability in HCT116 and SW480 cells, respectively. **(E)** Colony formation assay assessing the colony-forming ability of HCT116 and SW480 cells. **(F, G)** Wound healing assay of migration in HCT116 and SW480 cells, respectively. **(H, I)** Transwell assay of migration and invasion in HCT116 and SW480 cells, respectively. **(J)** Efficiency of RIPK2 knockdown in SW620 cells. **(K)** RIPK2 promotes CRC cell growth *in vivo*. (*p<0.05, **p<0.01, and ***p<0.001).

CRC is fundamentally characterized by the uncontrolled proliferation of cells (29). In our study, we considered the upregulated genes as crucial elements contributing to the malignant progression of CRC cells. Previous research has consistently linked these upregulated genes to processes involved in tumor growth and dissemination (30). However, despite existing reports associating RIPK2 with promoting malignant progression and poor prognosis in various solid tumors, its precise functions and regulatory mechanisms in CRC remain unclear. To bridge this knowledge gap, we obtained IHC results from the HPA database, revealing a significant upregulation of RIPK2 protein levels in CRC (**Figures 10A, B**). To delve deeper into the impact of RIPK2 on CRC cell proliferation, we established RIPK2 knockdown models using HCT116 and SW480 cells (**Figure 10C**). CCK-8 and colony formation assays collectively revealed a marked decrease in cell viability and colony-forming ability following RIPK2 knockdown in CRC cells (**Figures 10D, E**). The results demonstrated a significant reduction in migration and invasion abilities in the sh-RIPK2 group compared with the control group (**Figures 10F-I**). Collectively, these findings provide compelling evidence that RIPK2 actively promotes the malignant progression of CRC cells. To further illuminate the effects of RIPK2 on CRC, animal studies were conducted, showing a substantial reduction in volume and weight of xenograft tumors when RIPK2 was knocked down (**Figures 10J, K**).

## Discussion

CRC is one of the most common gastrointestinal malignancies with worldwide high morbidity and mortality (31). It is worth noting that the CRC TME (comprised of tumor cells, inflammatory immune cells, fibroblasts, and vascular cells) prevents immunosurveillance, which promotes tumor progression and metastasis (32). Further, the inflammatory response has a significant impact on the plasticity and functional characteristics of cancer and stromal cells within the TME (10), with either beneficial or adverse consequence for disease progression (33). Contextual evaluation of the TME, inflammation, and cancer is fundamental to an understanding of the molecular basis for CRC and to the design of new therapeutic targets for CRC.

Herein, we classified CRC subtypes based on DE-IRG expression profiles and evaluated relationships between the inflammatory response and CRC clinico-pathologic traits. Pathway enrichment analysis was used to compare TME inflammation-related subtypes. Further, ssGSEA, IPS algorithm, TMB, and drug sensitivity were analyzed to predict a patient’s therapeutic response to ICI and chemotherapy. In addition, we constructed a risk-scoring system consisting of 11 inflammation-related genes. Findings provided for a high degree of explanation and interpretation, yielding valuable insight into predictive prognosis and the identification of novel biomarkers and treatment modalities for CRC.

We classified patients with CRC into INF-high and INF-low groups; the latter was associated with a favorable prognosis based on survival analysis. Consistent with our study, unbalanced and unlimited chronic inflammation, as a consistent component of cancer, is associated with disease progression and a poor prognosis (34, 35). Analysis of clinico-pathological characteristics by this study demonstrated that more malignant and advanced CRC patients were found within the INF-high cluster. This cluster had the worse prognosis.

Herein, the immune microenvironment was analyzed to explore prognostic differences between the subtypes. Compared to the INF-low group, the INF-high group had a higher immune and estimate scores, suggesting abundant immune cell and non-tumor cell infiltration within the INF-high CRC patients. Tumor purity may reflect TME components and is negatively related to clinical outcomes, as described in previous studies (36–38). The INF-high subgroup had higher tumor purity and was associated with a poor prognosis. The mismatched immune infiltration and survival disadvantage for the INF-high subgroup may be due to the rare presence of T cells within tumor islets. A dense stromal matrix may restrain migration of T cells and thus weaken antitumor immunity (39). Higher stroma-related gene expression is associated with a poorer immunotherapy response and worse survival (40). We speculated that different components of the TME play paradoxical roles and as such we assessed immune cell infiltration.

Compared to the INF-low group, the INF-high group exhibited abundant immune cell infiltration including immunosuppressive cells such as Tregs, mast cells, and macrophages. Previous studies have reported that Tregs suppress antitumor immunity and promote tumor progression (41). Tregs and tumor-associated macrophages contribute to the creation of an immunosuppressive network, playing a key role in the development of tumor immune evasion (42–44). Considering the significant immunosuppressive roles of Tregs and macrophages, the abundant immune infiltration observed in INF-high does not guarantee enhanced tumor suppression and immune killing capabilities compared to INF-low. In addition, though tumor infiltrating lymphocytes (TILs) play pivotal roles in determining survival rates, when activated T-cells fail to completely eliminate the tumor, they can become exhausted over time (45). This exhaustion, a consequence of prolonged antigen exposure, results in cytotoxic T-cells becoming compromised, rendering them inefficient at performing their effector functions (46). Prior research has demonstrated that elevated levels of CTLs and a heightened incidence of T cell dysfunction which may lead to increased tumor immune evasion, through a more severe degree of T cell dysfunction (47). Such immune cell exhaustion, common in cancer and various other conditions, leads to malignant progression despite an otherwise operational immune system (48). TIDE, capable of quantifying immune dysfunction in tumors, reveals that INF-high subgroup, characterized by elevated TIDE scores, are more prone to exhibit T cell exhaustion (47). Together with the immunosuppressive cells, patients within the INF-high group were more likely to develop immune evasion.

Major histocompatibility complexes and chemokines were upregulated in the INF-high group, suggesting greater inflammatory activity (49). The chemokines CCL2 and CCL3 recruit pro-tumor macrophages into the TME (50). The chemokine receptors CXCR1, CXCR4, and CCR5 are associated with growth and metastasis of tumors (51–55). CXCL12 and CXCL8 have been linked to immunotherapy unresponsiveness for colon cancer and melanoma patients (56, 57). Thus, elevated chemokines within the INF-high subgroup were associated with disease progression and insensitivity to immunotherapy, resulting in poorer survival.

Advanced understanding of the pathophysiology of CRC, combined with a cumulative interest in immunotherapy and chemotherapy, have significantly increased the number of treatment options for CRC patients (31). Only 15% of CRC patients are MSI-H and benefit from immunotherapy. Most patients are MSS and have ‘cold tumors’ that are insensitive to ICI (58). It is urgent to find effective biomarkers that can predict whether a CRC patient would benefit from immunotherapy. In this study, the INF-high subgroup was positively related to TIDE score, which indirectly indicates that INF-high patients are less responsive to ICI and more likely to suffer immune evasion. The IPS score is valuable for prediction of cancer patient response to immunotherapy with anti-PD-1 and anti-CTLA-4 treatment (59). Levels of IPS and IPS-CTLA4 were lower for INF-high individuals, indicating that immune checkpoint inhibitors were less effective for treatment of INF-high patients, which may account for their poor clinical outcomes. Thus, although patients within the INF-high group present with high TMB and MSI-H, they respond poorly to ICI. According to theory, inflammation induced by specific treatments may accelerate tumor progression and block the efficacy of anti-PD-1 therapy (60). Furthermore, the IC50 values for key drugs, such as 5-FU and Irinotecan, are significantly higher in patients with INF-low than in those with INF-high. This suggests that CRC patients exhibiting a high inflammatory response may exhibit increased sensitivity to common chemotherapeutic agents including 5-Fluorouracil, Irinotecan, Camptothecin, and Docetaxel. These drugs primarily target pathways related to DNA or RNA synthesis and cell division, aiming to inhibit cancer cell proliferation. In addition, we have identified non-traditional drugs—Lapatinib, Selumetinib, Vorinostat, and Gefitinib—that may offer a therapeutic advantage for INF-low patients who might not respond to conventional treatments. These drugs specifically target signaling pathways or receptors associated with tumor growth and survival. They have the potential to modulate receptor tyrosine kinases, MAPK/ERK signaling, or histone deacetylases, thereby impeding tumor progression.

Finally, to streamline inspection and identification of patients at high risk for early intervention, we constructed a risk model based on 11 prognostic signatures identified by machine learning methods, six of which (*CCL2, CMKLR1, NOD2, SLC28A2, SLAMF1, SRI*) were protective factors, and five of which (*MARCO, P2RX4, RGS16, RIPK2, VIP*) were related to high risk. Risk score was negatively associated with clinical outcomes. Results from single and multiple-factor Cox regression analysis demonstrated IRRS to be an independent prognostic marker for CRC. We constructed a nomogram by combining risk score and other independent prognostic factors, which had superior predictive value and higher net benefit than risk score.

To effectively predict survival, we screened two inflammation-related genes (*RIPK2* and *NOD2*) that were highly expressed in cancer tissues and which influenced clinical outcomes. *RIPK2* and *NOD2* (included in the model) are associated with the occurrence and progression of IBD, affecting intestinal homeostasis (61, 62). As an intracellular pathogen pathway, *NOD2/RIPK2* is a molecular driver of tumorigenic inflammation. *RIPK2* is an intermediate of *NOD2* signaling. During the transformation from acute to chronic inflammation, hyperactive *RIPK2* plays an important role in abnormal inflammation, altered metabolism, cell proliferation, and tumorigenesis (63). *NOD2* controls intestinal inflammation thereby decreasing the incidence of inflammatory driven cancers, including CRC, by downregulation of TLR pathways (64, 65).

This investigation is limited in that the study cohorts were collected from different platforms and different public datasets. Intra-tumor and intra-patient heterogeneity was a limitation, with future analysis benefiting from greater calibration. As well, this study and other similar studies are limited by the general lack of CRC patient data. Further, although identified clusters appeared to have a strong power for prognostic assessment, the cellular and molecular mechanisms underpinning the assessment is unclear. *In vivo* and *in vitro* biological experiments are required to verify the role of inflammation pathways in CRC. As well, the reliability and repeatability of the predictive model require further validation with other datasets and prospective as well as multi-center large-sample studies. Finally, factors included in the nomogram may have been inappropriate in that initiation and risk factors for CRC are not well defined. With further definition a more accurate nomogram may be built by incorporation of new risk factors and clinical elements.

In conclusion, by combining bioinformatics analysis and *in vitro* experiments, we have identified a new CRC molecular subtype based on the inflammatory response. Results demonstrated the inflammation related model to be a credible prognostic approach for CRC patients through analysis of immune cell infiltration and immunotherapy response. The inflammation-high subtype is associated with a dismal prognosis, an immunosuppressive microenvironment, and more advanced malignant tumors. In contrast, the inflammation-low subtype was associated with favorable clinical outcomes and an immune-reactive microenvironment. The inflammation related model may provide improved predictive prognosis for CRC patients and may be useful as a means to accurately stratify CRC patients. However, it is important to note that the interplay between inflammation markers and the immune microenvironment requires further investigation. In summary, this bioinformatics study serves as a foundation for understanding the role of IRGs in CRC.

## Conclusion

In conclusion, we identified two distinct inflammation related clusters based on DE-IRGs and developed an inflammation related risk score model for colorectal cancer, which can reveal the relationship between inflammation and tumor immune environment and provide references for early intervention and individualized treatment of colorectal cancer.

## Data availability statement

The original contributions presented in the study are included in the article/**Supplementary Material**. Further inquiries can be directed to the corresponding authors.

## Ethics statement

The studies involving humans were approved by the Ethics Committees of Peking University Cancer Hospital & Institute (Beijing, China). The studies were conducted in accordance with the local legislation and institutional requirements. The participants provided their written informed consent to participate in this study. The animal study was approved by The animal experiments were approved by the Animal Ethics Committee of Peking University Cancer Hospital & Institute. The study was conducted in accordance with the local legislation and institutional requirements.

## Author contributions

JP: Conceptualization, Data curation, Formal analysis, Funding acquisition, Investigation, Methodology, Project administration, Resources, Software, Supervision, Validation, Visualization, Writing – original draft, Writing – review & editing. YG: Conceptualization, Data curation, Formal analysis, Funding acquisition, Investigation, Methodology, Project administration, Resources, Software, Supervision, Validation, Visualization, Writing – original draft, Writing – review & editing. AW: Conceptualization, Data curation, Formal analysis, Funding acquisition, Investigation, Methodology, Project administration, Resources, Software, Supervision, Validation, Visualization, Writing – original draft, Writing – review & editing.
