# Supplementary material for: An inflammation-related subtype classification for analyzing tumor microenvironment and clinical prognosis in colorectal cancer
Source: Front Immunol. 2024 Apr 29;15:1369726. doi: 10.3389/fimmu.2024.1369726 (PMC11089903; doi:10.3389/fimmu.2024.1369726)
Supplement: Supplementary file 1 [file Presentation_1.pdf]

Supplementary Materials

1.1 Supplementary Methods

Cell culture and immunohistochemistry (IHC)

Human CRC cell lines (HCT116, SW480, and SW620) were procured from the American Type Culture Collection (Manassas, VA, USA). The cells were cultured in RPMI medium (Gibco, Carlsbad, CA, USA) or DMEM medium (Gibco, Carlsbad, CA, USA), both of which were supplemented with 10% fetal bovine serum (FBS) in a humidified incubator at 37 °C in the presence of 5% CO2. Regular passage of cells was performed, and routine checks for mycoplasma infection were conducted. Cells could be used only when the test result was negative. To assess the protein expression levels of RIPK2 between CRC and normal samples, IHC data and images obtained from the Human Protein Atlas (HPA, https://www.proteinatlas.org/) were applied for analysis and comparison.

Cell transfection

To construct stable RIPK2 knockdown cell lines, recombinant lentiviruses expressing three shRNAs targeting RIPK2 and negative control (LV-shNC) were designed and cloned into GV248 vector by Genomeditech (Shanghai, China). CRC cells were selected by puromycin for 2 weeks after 72h infection with lentiviruses at a multiplicity of infection of 30 for SW480 and 10 for HCT116 and SW620. The shRNA sequences used in this research are listed in Supplementary Table S3.

1.2 Supplementary Tables

Table 1. Catalog of Inflammation-Related Genes.

Gene Name
ABCA1 ABI1 ACVR1B ACVR2A ADM ADORA2B ADRM1 AHR APLNR AQP9 ATP2A2 ATP2B1 ATP2C1 AXL BDKRB1 BEST1 BST2 BTG2 C3AR1 C5AR1 CALCRL CCL17 CCL2 CCL20 CCL22 CCL24 CCL5 CCL7 CCR7 CCRL2 CD14 CD40 CD48 CD55 CD69 CD70 CD82 CDKN1A CHST2 CLEC5A CMKLR1 CSF1 CSF3 CSF3R CX3CL1 CXCL10 CXCL11 CXCL6 CXCL9 CXCR6 CYBB DCBLD2 EBI3 EDN1 EIF2AK2 EMP3 ADGRE1 EREG F3 FFAR2 FPR1 FZD5 GABBR1 GCH1 GNA15 GNAI3 GP1BA GPC3 GPR132 GPR183 HAS2 HBEGF HIF1A HPN HRH1 ICAM1 ICAM4 ICOSLG IFITM1 IFNAR1 IFNGR2 IL10 IL10RA IL12B IL15 IL15RA IL18 IL18R1 IL18RAP IL1A IL1B IL1R1 IL2RB IL4R IL6 IL7R CXCL8 INHBA IRAK2 IRF1 IRF7 ITGA5 ITGB3 ITGB8 KCNA3 KCNJ2 KCNMB2 KIF1B KLF6 LAMP3 LCK LCP2 LDLR LIF LPAR1 LTA LY6E LYN MARCO MEFV MEP1A MET MMP14 MSR1 MXD1 MYC NAMPT NDP NFKB1 NFKBIA NLRP3 NMI NMUR1 NOD2 NPFFR2 OLR1 OPRK1 OSM OSMR P2RX4 P2RX7 P2RY2 PCDH7 PDE4B PDPN PIK3R5 PLAUR PROK2 PSEN1 PTAFR PTGER2 PTGER4 PTGIR PTPRE PVR RAF1 RASGRP1 RELA RGS1 RGS16 RHOG RIPK2 RNF144B ROS1 RTP4 SCARF1 SCN1B SELE SELL SELENOS SEMA4D SERPINE1 SGMS2 SLAMF1 SLC11A2 SLC1A2 SLC28A2 SLC31A1 SLC31A2 SLC4A4 SLC7A1 SLC7A2 SPHK1 SRI STAB1 TACR1 TACR3 TAPBP TIMP1 TLR1 TLR2 TLR3 TNFAIP6 TNFRSF1B TNFRSF9 TNFSF10 TNFSF15 TNFSF9 TPBG VIP

Table 2. Primers sequences used in qRT-PCR.

Gene	Forward primer (5’ → 3’)	Reverse primer (5’ → 3’)
NOD2	CACGGTGAAAGCGAATGGATTGG	GAGGAAGCGAGACTGAGCAGAC
RIPK2	TTAACCAGTCGCTAGATGCCCTTC	AGGTCCTTGTAGGCTTGGTACTAAC
GAPDH	CTCAGACACCATGGGGAAGGTGA	ATGATCTT2GAGGCTGTTGTCATA

Table 3. Sequences of shRNAs for RIPK2.

shRNA	Sequence
RIPK2 shRNA1	5'-GCCAGTATCAAGCACGATATA-3'
RIPK2 shRNA2	5'-GCACCATTTCTGGATCTCAAA-3'
RIPK2 shRNA3	5'-CACCAATCCTTTGCAGATAAT-3'
Negative control sense	5'-TTCTCCGAACGTGTCACGT-3'

1.3 Supplementary Figures

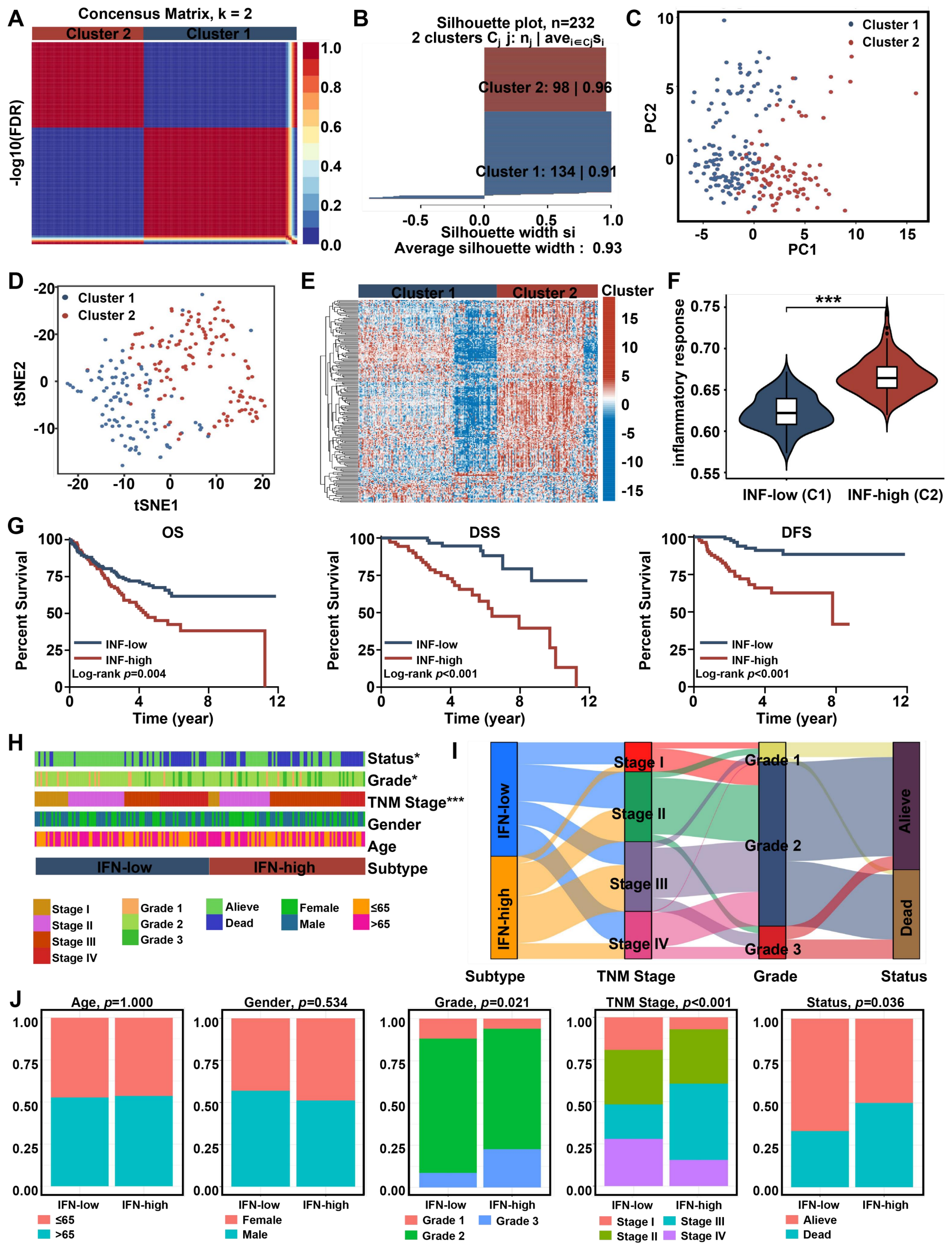

Supplementary Figure 1. Consensus clustering of inflammation-related genes in validation cohort. (A) The consensus clustering heat map visualizes the degree of segmentation. (B) The average silhouette width represents the coherence of clusters. (C) Principal component analysis plots. (D) tSNE plots. (E) Heatmap of 65 inflammation related genes expression in different subgroups; red represents high expression, and blue represents low expression. (F) Violin plots indicating the differences in these subtypes. (G) Kaplan-Meier overall survival, disease-specific survival, and disease-free survival curves. (H) Heatmap presenting the clinicopathologic features of these subtypes. (I) Sankey diagram showing the relationship between inflammation subtype, MSI status, T stage, cancer type, TNM stage and status. (J) The distribution characteristics of different clinicopathological factors in two subtypes. (*p<0.05, **p<0.01, and ***p<0.001).

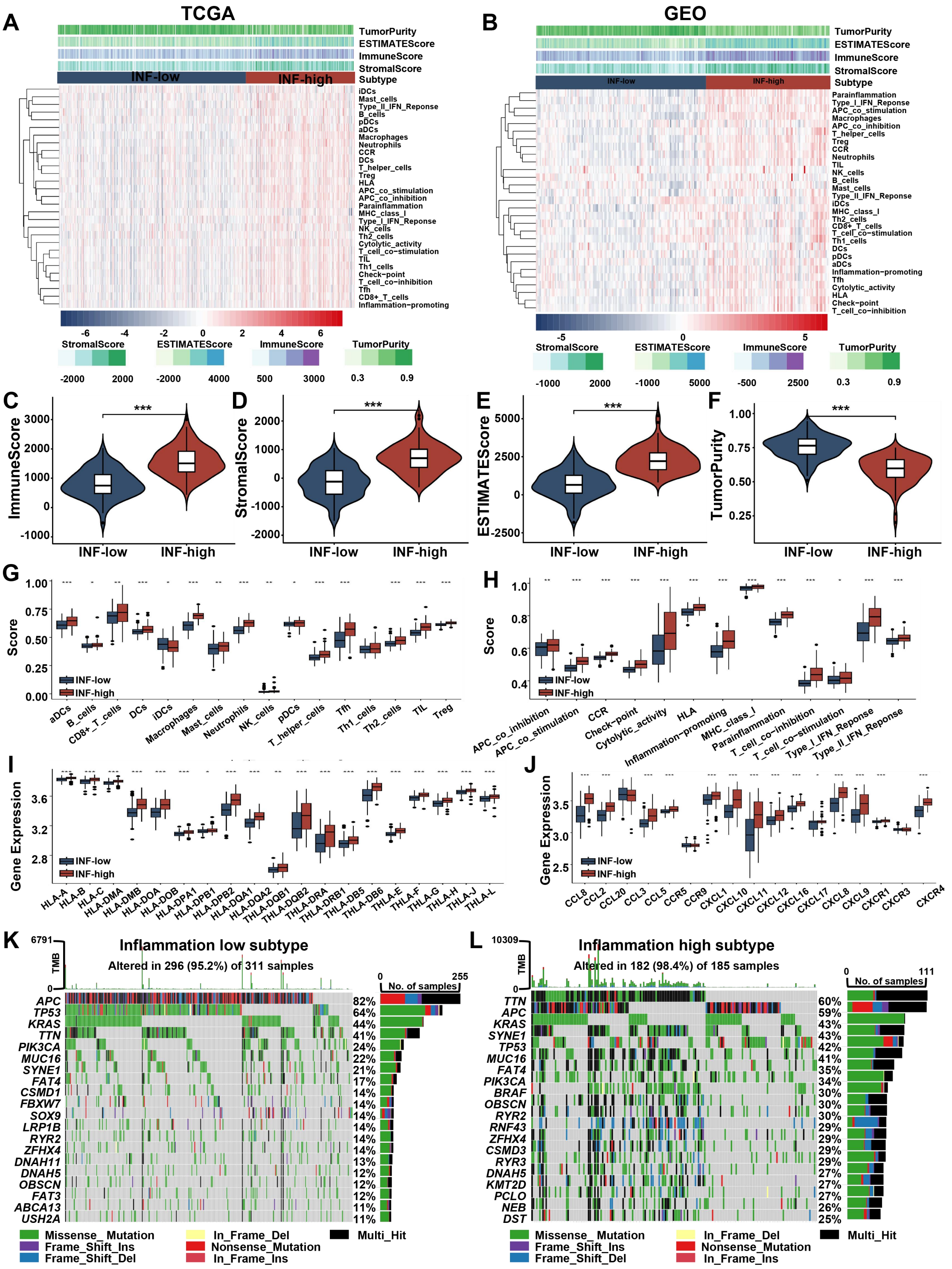

Supplementary Figure 2. Analysis of tumor microenvironment (TME) landscape and somatic mutation between the two subtypes. (A-B) The heatmaps show 29 immune-related gene sets, immune score, stromal score, ESTIMATE score, and tumor purity in the training cohort and validation cohort. (C-F) The violin plots display the immune score, stromal score, estimate score, and tumor purity score in the training cohort. (G-J) Boxplots representing the differential expression of immune cells, immune cell subpopulations related functions, HLA gene sets and chemokines. (K-L) Somatic mutations in the training cohort and validation cohort.(*,<0.05; **,<0.01; ***<0.001)

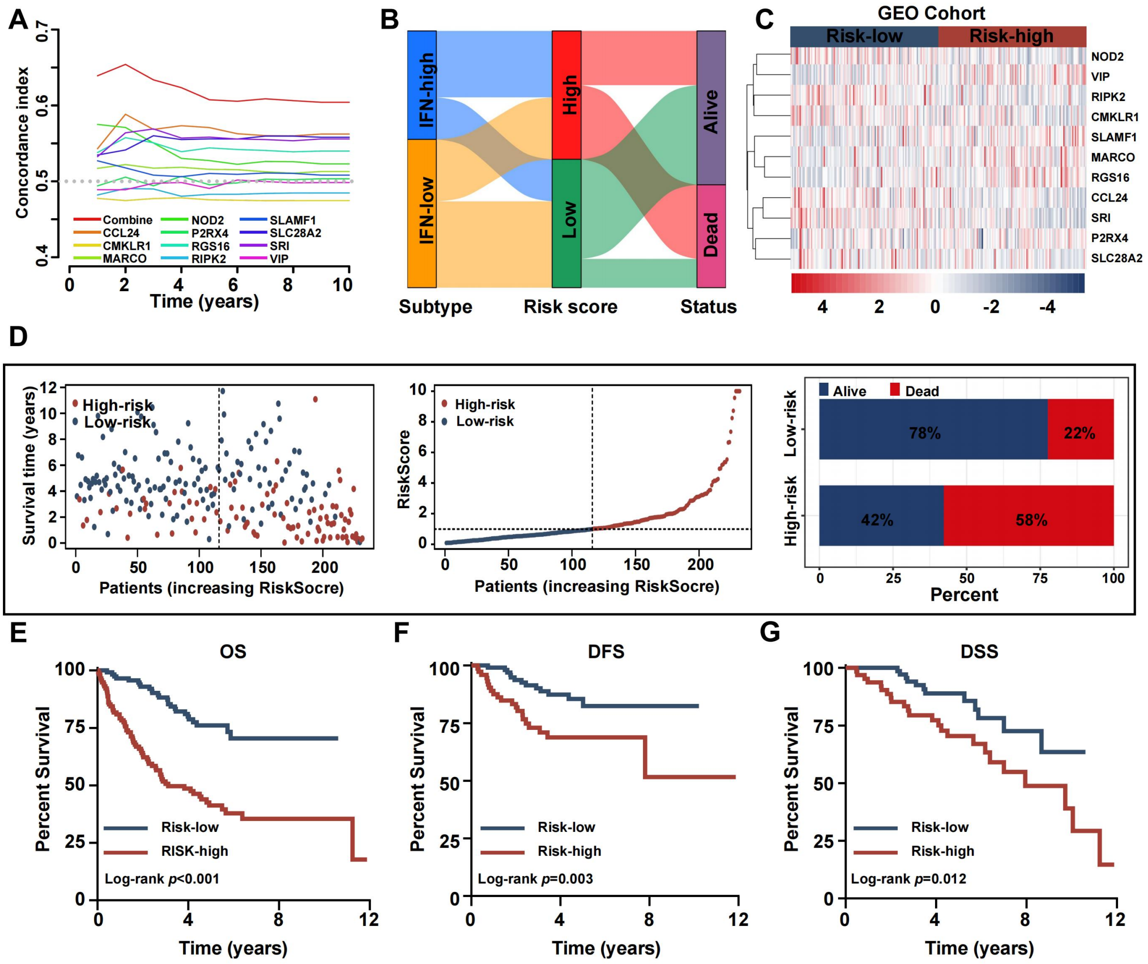

Supplementary Figure 3. Construction and validation of the inflammation-related prognostic signature in validation cohort. (A)Time-dependant C-index plot for the risk score and individual genes. (B) Sankey plot summarized the relationships among the clusters, IRRS and survival status. (C-D) Heatmaps of prognostic eleven-gene risk signature, risk scores distribution, and survival status of each patient. (E-G) Kaplan-Meier overall survival, disease-free survival curves, and disease-specific survival for patients with high- or low-risk scores in GEO validation cohort.
